# Prion Replication Occurs in Endogenous Adult Neural Stem Cells and Alters Their Neuronal Fate: Involvement of Endogenous Neural Stem Cells in Prion Diseases

**DOI:** 10.1371/journal.ppat.1003485

**Published:** 2013-08-01

**Authors:** Aroa Relaño-Ginès, Audrey Gabelle, Claire Hamela, Maxime Belondrade, Danielle Casanova, Chantal Mourton-Gilles, Sylvain Lehmann, Carole Crozet

**Affiliations:** 1 Institut de Génétique Humaine, CNRS-UPR 1142, Montpellier, France; 2 Institut de Recherche en Biothérapie (IRB), Physiopathologie, Diagnostic et Thérapie Cellulaire des Affections Neurodégénératives - INSERM-UM1 U1040, CHU de Montpellier, Université Montpellier 1, Montpellier, France; 3 ANSM, 635, Vendargues, France; University of Edinburgh, United Kingdom

## Abstract

Prion diseases are irreversible progressive neurodegenerative diseases, leading to severe incapacity and death. They are characterized in the brain by prion amyloid deposits, vacuolisation, astrocytosis, neuronal degeneration, and by cognitive, behavioural and physical impairments. There is no treatment for these disorders and stem cell therapy therefore represents an interesting new approach. Gains could not only result from the cell transplantation, but also from the stimulation of endogenous neural stem cells (NSC) or by the combination of both approaches. However, the development of such strategies requires a detailed knowledge of the pathology, particularly concerning the status of the adult neurogenesis and endogenous NSC during the development of the disease. During the past decade, several studies have consistently shown that NSC reside in the adult mammalian central nervous system (CNS) and that adult neurogenesis occurs throughout the adulthood in the subventricular zone of the lateral ventricle or the Dentate Gyrus of the hippocampus. Adult NSC are believed to constitute a reservoir for neuronal replacement during normal cell turnover or after brain injury. However, the activation of this system does not fully compensate the neuronal loss that occurs during neurodegenerative diseases and could even contribute to the disease progression. We investigated here the status of these cells during the development of prion disorders. We were able to show that NSC accumulate and replicate prions. Importantly, this resulted in the alteration of their neuronal fate which then represents a new pathologic event that might underlie the rapid progression of the disease.

## Author Summary

Prion diseases are irreversible progressive neurodegenerative diseases, leading to severe incapacity and death. They are considered to be caused by an abnormally folded infectious protein named PrP^Sc^. They are characterized in the brain by prion amyloid deposits, vacuolisation, astrocyte proliferation, neuronal degeneration, and by cognitive, behavioural and physical impairments. There is no treatment for these disorders. Transplantation of stem cells, or the stimulation of endogenous neural stem cells (NSC) in the adult brain therefore constitute new interesting and promising strategies. While our first interest was the development of a cell therapy approach, we rapidly realised that there were a lot of questions to address before investigating pre-clinical cell therapy assays. Some of them were: (i) what is the status of endogenous neural stem cells during the development of prion diseases and can they amplify prions, and (ii) can they still proliferate and give rise to new neurons. In this study we definitely demonstrate that PrP^Sc^ was not only able to replicate in adult neural stem cells derived from infected brains but also that this results in an impairment of the production of their neuronal derivatives.

## Introduction

Prion diseases or transmissible spongiform encephalopathies (TSEs) are fatal neurodegenerative disorders, which include Creutzfeldt-Jakob disease in humans, scrapie in sheep and goats, and bovine spongiform encephalopathy in cattle. Their origin can be genetic, sporadic or infectious and there is currently no available treatment preventing the widespread neurodegeneration occurring in these disorders. TSEs are pathophysiologically characterized by the accumulation in the brain of a pathogenic abnormal isoform of a protein termed PrP scrapie (PrP^Sc^) [1]. According to the prion hypothesis, the infectious isoform PrP^Sc^ can trigger the autocatalytic conversion of the neuronal host-encoded PrP^C^ into PrP^Sc^ [2] through a poorly understood misfolding process [1], rendering the progression of the disease dependent upon PrP expression. Several studies have reported early, severe and selective loss of GABAergic interneurons in prion diseases [3], [4]. These specific changes in neuronal subset may underlie some of the clinical symptoms in prions. The diagnosis of these diseases is difficult and often leaves only a short therapeutic window after the appearance of the first clinical signs [5]. Although important efforts have been made to understand the physiopathogenesis of neurodegenerative disorders, Prion diseases are still incurable and new therapeutic approaches such as cell therapy need to be explored. As a matter of fact, the widespread existence of endogenous neural stem cells (NSC) in the adult brain [6], [7] offers hope that these endogenous cells may be harnessed to repair cellular damages caused by brain injuries. During the past decade, several studies have consistently shown that (i) NSC reside in the adult mammalian CNS and that (ii) adult neurogenesis occurs throughout the adulthood in the subventricular zone (SVZ) of the lateral ventricle (LV) or the Dentate Gyrus (DG) of the hippocampus (H). Accumulating evidences have clearly shown that a large number of newborn neurons can be generated from adult NSC, and integrate into pre-existing neural circuits [8]. Under physiological conditions, adult NSC follow a highly stereotypic differentiation path to generate neurons in the olfactory bulb and the DG. Adult neurogenesis is also highly sensitive to environmental cues, physiological stimuli and neuronal activity, suggesting that the tailored addition of new neurons might serve specific neuronal functions [9]. Endogenous NSC may also provide a cellular reservoir for replacement of cell lost during normal cell turnover but also after brain injury [10], [11]. In neurodegenerative affections, particularly those involving pathogenic protein misfolding, the field of adult neurogenesis only begins to be explored. The results are not always consistent between studies. For instance, hippocampal neurogenesis is increased in patients with AD [12], but it is decreased in some transgenic mouse models of AD [13]. Following brain injuries, adult neurogenesis can be increased and is even accompanied by a migration of neural precursors towards the injured area [13], [14]. However, the activation of this system does not fully compensate the neuronal loss that occurs during diseases and could even contribute to the disease progression [15].

In prion diseases, while it has been suggested that adult neurogenesis was increased [16], the role and the status of adult NSC are still obscure. Despite the fact that we were able to propagate prions in vitro in NSC from fetal [17] or adult origin, we did not know whether endogenous NSC also accumulated prion in vivo and the impact this would have. This question has been addressed in this study and we showed that endogenous NSC were not only infected by prions but also that their neuronal differentiation process was altered.

## Results

### PrP^Sc^ accumulation in NSC and neuroblasts area in prion infected mice

**Figure 1:**
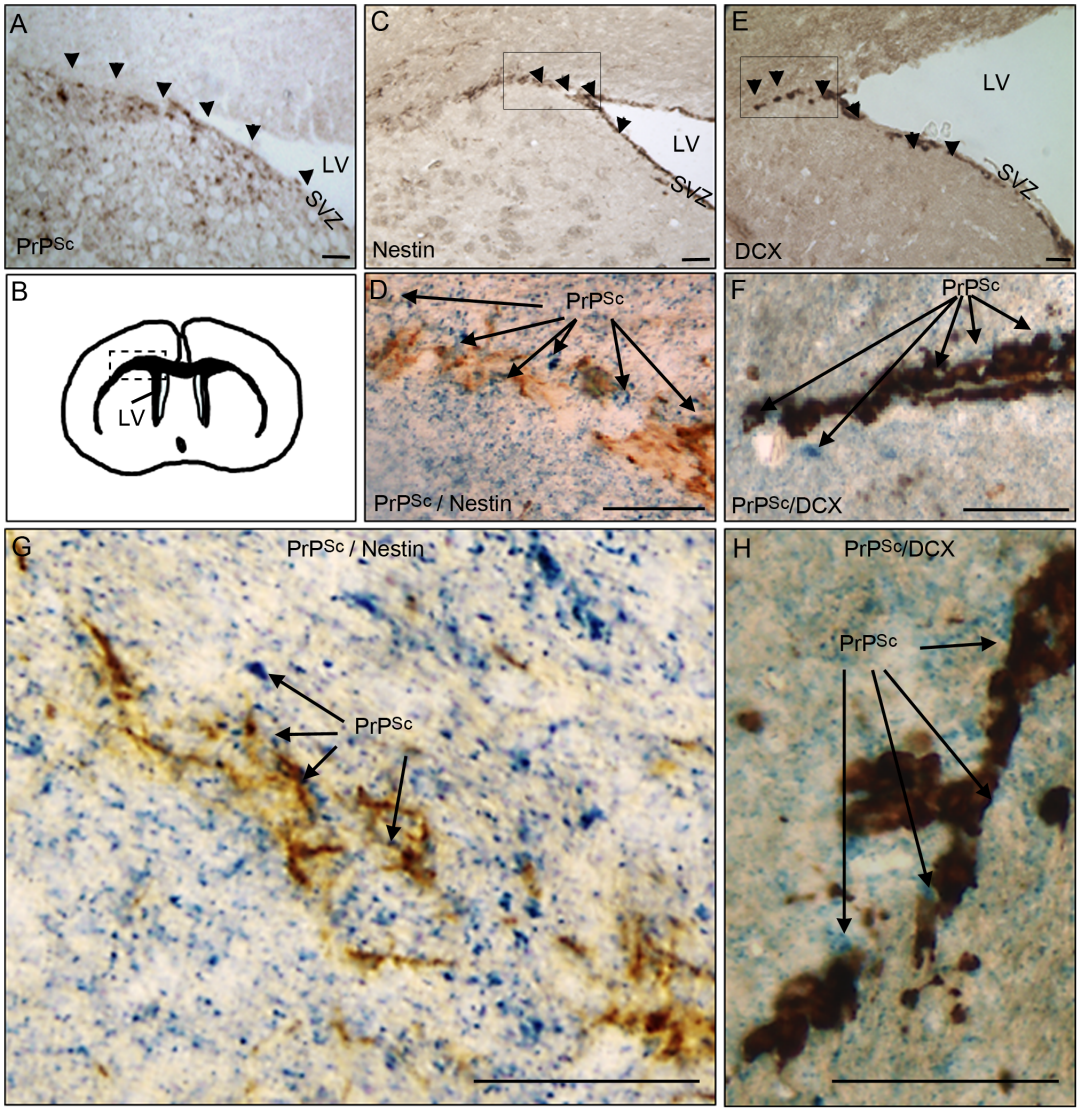
PrP^Sc^ is present in NSC and neuroblasts areas. **A**. PrP^Sc^ immunostaining (brown+arrow) using Saf84 anti-PrP antibody in the lateral wall (SVZ) and the surrounding of the lateral ventricle (LV). **B**. Schematic representation of the LV localisation in the mouse brain. **C**. Nestin immunostaining (brown+arrow) in the SVZ. **D and G**. Double-immunostaining of PrP^Sc^ (Blue) and Nestin (Brown) in the surrounding of the LV. **E**. Doublecortin (DCX) immunostaining (brown+arrow) showing neuroblasts exiting the SVZ of the lateral ventricle. **F and H** Double-immunostaining of PrP^Sc^ (Blue) and DCX (Brown) in the surrounding of the LV. Scale bar 20 µm.

In order to investigate whether pathological prion protein (PrP^Sc^) deposits were present in brain area containing adult neural stem cells and/or their derivative neuroblasts, we first performed immunohistochemical analyses of PrP^Sc^, nestin and doublecortin in mice that have been intracerebrally infected with the ME7 prion strain. Nestin is a NSC marker and doublecortin (DCX) is a neuroblast and immature neurons marker. The PrP^Sc^ deposits were densely present in the DG and LV neuronal progenitor area as well as in the DCX neuroblasts area surrounding the LV in mouse infected brain at the endpoint of the disease (Figure 1A, B, C, E). The double immunohistochemical analysis of the nestin or DCX markers with PrP^Sc^ clearly confirmed that PrP^Sc^ deposits were present around nestin and DCX positive cells (Figure 1D, F, G, H).

### NSC isolated from prion infected mice accumulate PrP^Sc^

**Figure 2:**
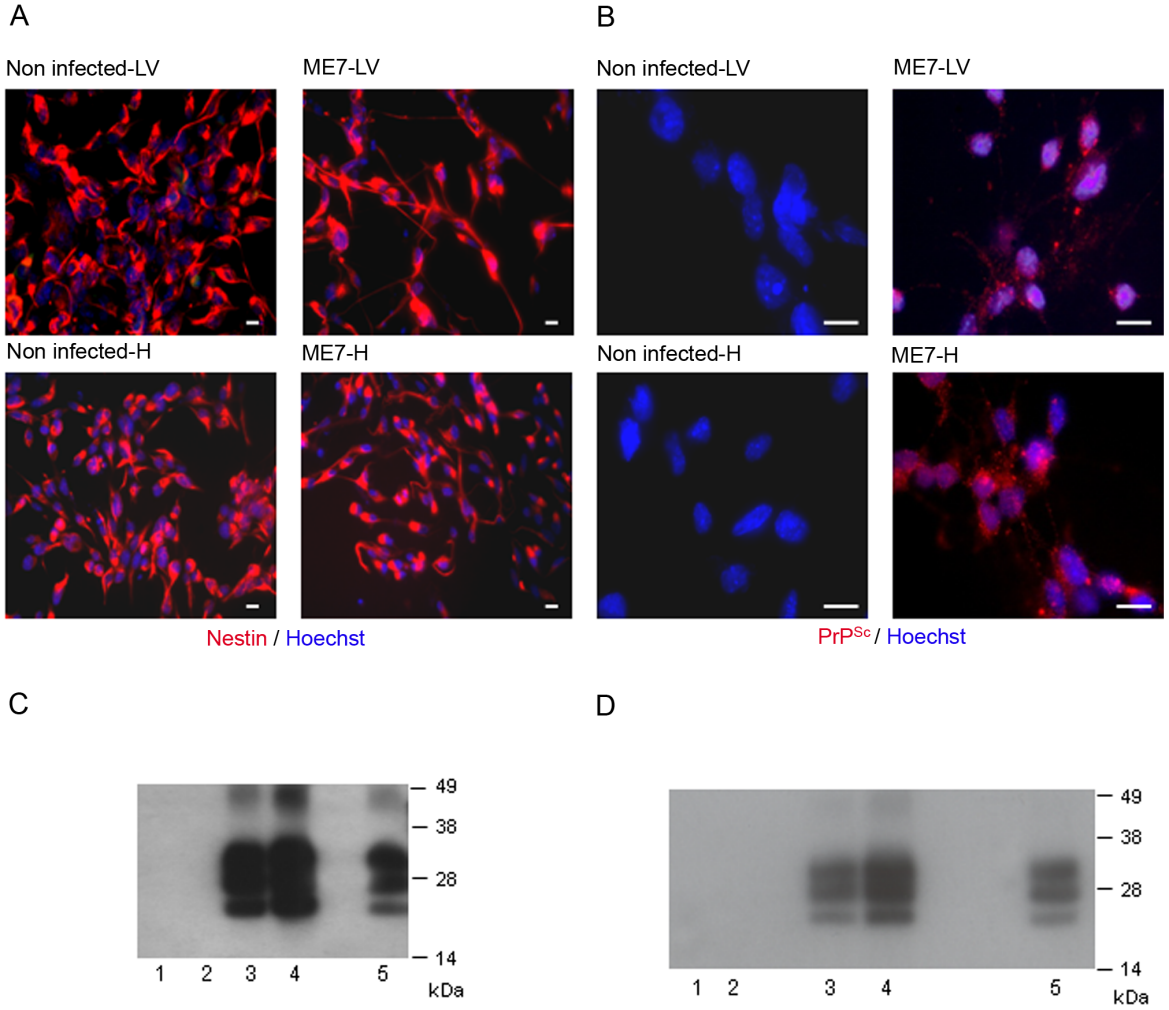
Neural precursor cells isolated from infected mice accumulate PrP^Sc^. **A**. Immunofluorescence analysis of the nestin marker in proliferative NSC derived from the hippocampus (H) or lateral ventricle (LV) of mock or ME7 infected mice. (red: nestin, Blue: Hoechst nuclei coloration, scale bar 5 µm). Most of the cells are positive for the nestin NSC marker in proliferation conditions. **B**. PrP^Sc^ Immunofluorescence in NSC cells derived from the lateral ventricle and the hippocampus of mock or ME7 infected mice (red: PrP^Sc^ immunofluorescence using saf61 anti-PrP antibodies, Blue: Hoechst nuclei coloration, scale bar 5 µm). **C**. PrP^Sc^ Western blot using Saf-Mix anti-PrP antibody: PK digested cell lysates of NSC cells derived from the lateral ventricle (1) and the hippocampus (2) of non infected mice or the lateral ventricle (3) and the hippocampus (4) of ME7 infected mice after 2 subpassages. (5) PrP^Sc^ control from ME7 infected brain. Lanes without numbers were left empty on purpose. **D**. PrP^Sc^ Western blot using Saf-Mix anti-PrP antibody: PK digested cell lysates of NSC cells derived from the lateral ventricle (1) and the hippocampus (2) of non infected mice or the lateral ventricle (3) and the hippocampus (4) of ME7 infected mice after 15 subpassages. (5) PrP^Sc^ control from ME7 infected brain. Lanes without numbers were left empty on purpose.

We then aimed to determine whether endogenous NSC had been infected during the disease development. Adult NSC were therefore isolated from the hippocampus and the lateral ventricle of 10 mock and 10 ME7 prion inoculated mice, at 130 days post-infection (dpi). As expected, the cells cultivated under neurosphere free floating conditions were all, positive for the nestin NSC marker (Figure 2A). We showed after two subpassages (30 days after isolation) that only NSC derived from ME7 infected mice were positive for PrP^Sc^ accumulation as assessed by both immunofluorescence (Figure 2B) and western blot after Proteinase K digestion (Figure 2C). This PrP^Sc^ generation in adult NSC was shown to be stable since PrP^Sc^ could still be detected after 15 subpassages (Figure 2D).

**Figure 3:**
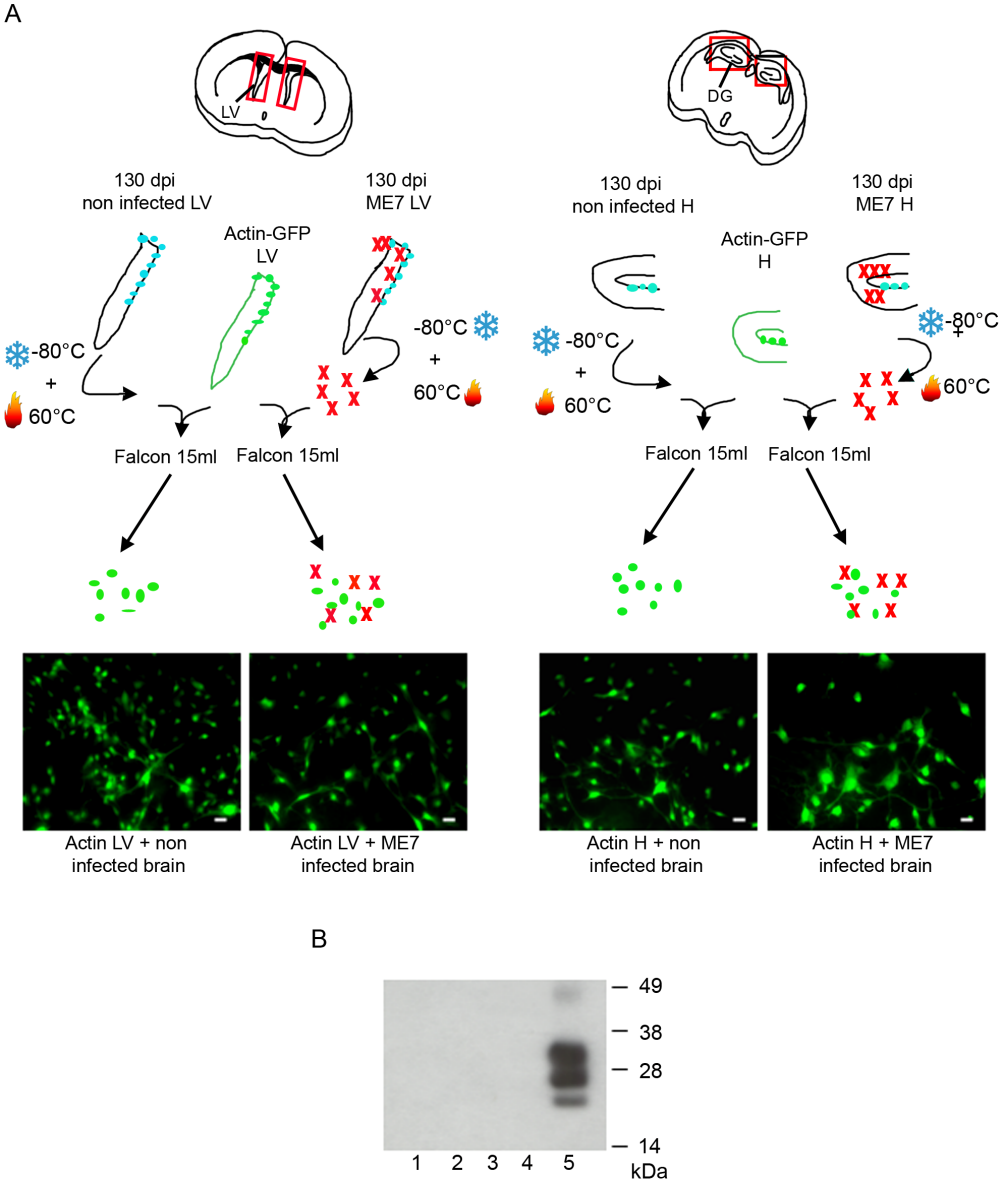
NSC were infected before their isolation. **A**. Schematic presentation of the experiment. Hippocampus and Lateral Ventricles were isolated from actin-GFP mice. An equivalent amount (weight) of non infected or infected tissue (in which cells have been frozen and then heat inactivated) was added in the same tube. The cells obtained were all positive for the GFP marker. **B**. PrP^Sc^ Western blot using Saf-Mix anti-PrP antibody: PK digested cell lysates of NSC cells derived from the lateral ventricle (1) and the hippocampus (2) of actin-GFP mice isolated in the presence of ME7, or from the lateral ventricle (3) and the hippocampus (4) of actin-GFP mice isolated in the presence of non infected brain samples after 2 subpassages. (5) PrP^Sc^ control from ME7 infected brain.

A thorough control experiment was then designed to confirm that the isolated cells were endogenously infected before the derivation and not during the cell culture. It consisted in the derivation of adult NSC from actin-GFP mice in the presence of an equivalent amount of a 130 dpi ME7 infected brain tissue. To avoid non-actin-GFP cells to proliferate, and therefore obtain only actin-GFP-NSC, the infected tissue dissected in each neurogenesis area was successively frozen at −80°C and heated at +60°C (Figure 3A). This procedure kills all the cells in the extract from non actin-GFP mice. In this paradigm, the derived actin-GFP NSC were not positive for PrP^Sc^ (Figure 3B) indicating that, in our experimental conditions, the PrP^Sc^ particles present in a 130 days infected brain were not sufficient to infect NSC.

### PrP^Sc^ accumulation in adult NSC impaired their neuronal differentiation

**Figure 4:**
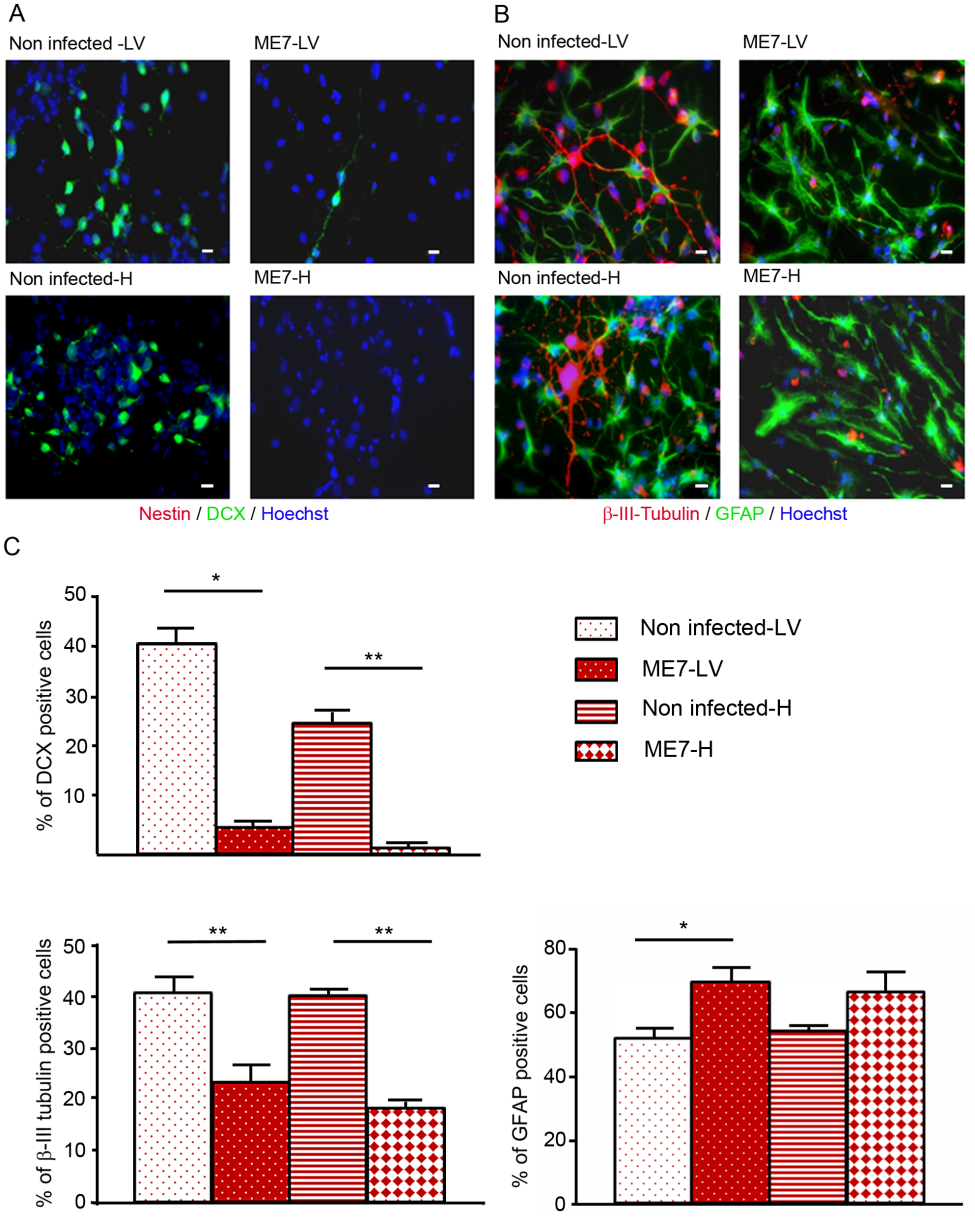
Impairment of the neuronal differentiation of NSC derived from prion infected mice. Immunofluorescence analysis of nestin, DCX, BetaIII-Tubulin and GFAP markers in differentiated NSC derived from the hippocampus (H) or Lateral ventricle (LV) of ME7 infected or non infected mice. (**A**. red: nestin; green: DCX, **B**. green: GFAP, red: BetaIII-Tubulin, blue: Hoechst nuclei coloration, scale bar 5 µm). **C**. Quantification of DCX and beta-III-Tubulin positive cells using ImageJ software. After differentiation induction, NSC gave rise to significantly more DCX positive cells in non infected conditions when compared with ME7 infected conditions (p<0.05 and p<0.01 by Mann-Whitney test). The number of neurons also differed significantly (p<0.01 by Mann-Whitney test) between non infected and infected NSC derived cells in differentiation conditions. Inversely astrocyte proportions were higher in the ME7 infected context (p<0.05 by Mann-Whitney test). Data represent means +/− SEM from one experiment performed in triplicate. Similar results were obtained in the three independent experiments.

The differentiation potential of these different NSC isolated from mock and prion infected mice was then analysed. To avoid a massive anoikis which is a ROCK signalling induced apoptosis that occurs after neurosphere dissociation [18], neurospheres were gently trypsinized and put on polyornithine/laminine coated dishes for one passage before being seeded for differentiation studies. To induce neuronal and glial differentiation, NSC were placed in a differentiation medium during 5 days. In these conditions, the nestin markers completely disappeared (Figure 4A) and NSC gave rise to neuroblasts (Figure 4A), young neurons and astrocytes (Figure 4B). Counting analyses of the number of DCX positive cells and betaIII-Tubulin positive cells were performed and are presented in Figure 4 (Fig. 4A, B, C). DCX positive neuroblasts (5% and less than 1% for ME7 derived LV-NSC and H-NSC instead of 40% and 25% for non infected derived LV-NSC and H-NSC) and BetaIII-Tubulin positive new born neurons (23.5% and 18% for ME7 infected LV-NSC and H-NSC instead of 41% and 40% for non infected LV-NSC and H-NSC) were less numerous (p<0.05 and p<0.01 respectively, MannWhitney Test) when cells were infected, indicating a defect in neuronal differentiation. Inversely, the proportion of astrocytes was higher in the ME7 infected cells (Figure 3C).

### Neuronal impairment takes place during the differentiation process

**Figure 5:**
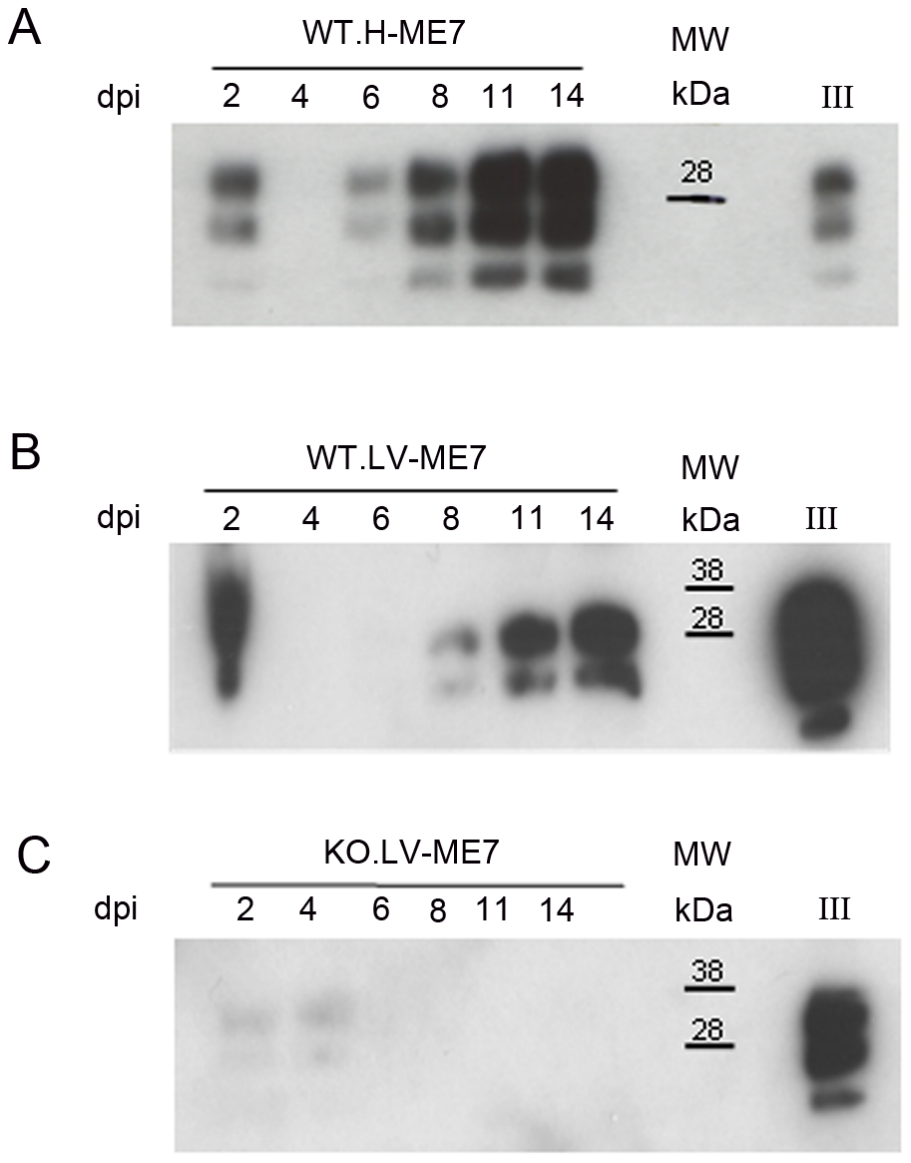
Infection of NSC during differentiation. Western blots showing the generation of PrP^Sc^: (A) in hippocampus derived NSC, (B) in lateral ventricle derived NSC, (C) in KOPrP LV derived NSC, during the differentiation process and after incubation with ME7 brain homogenate at the time of the differentiation induction. All the samples were treated with PK, Saf Mix cocktail of anti-PrP antibodies was used to detect proteinase K resistant PrP^Sc^. Cells were harvested at 2, 4, 6, 8, 11 and 14 dpi, after brain homogenate removal. III: Positive control ME7, MW : Molecular Weight (in kDa). Lanes without numbers were left empty on purpose.

**Figure 6:**
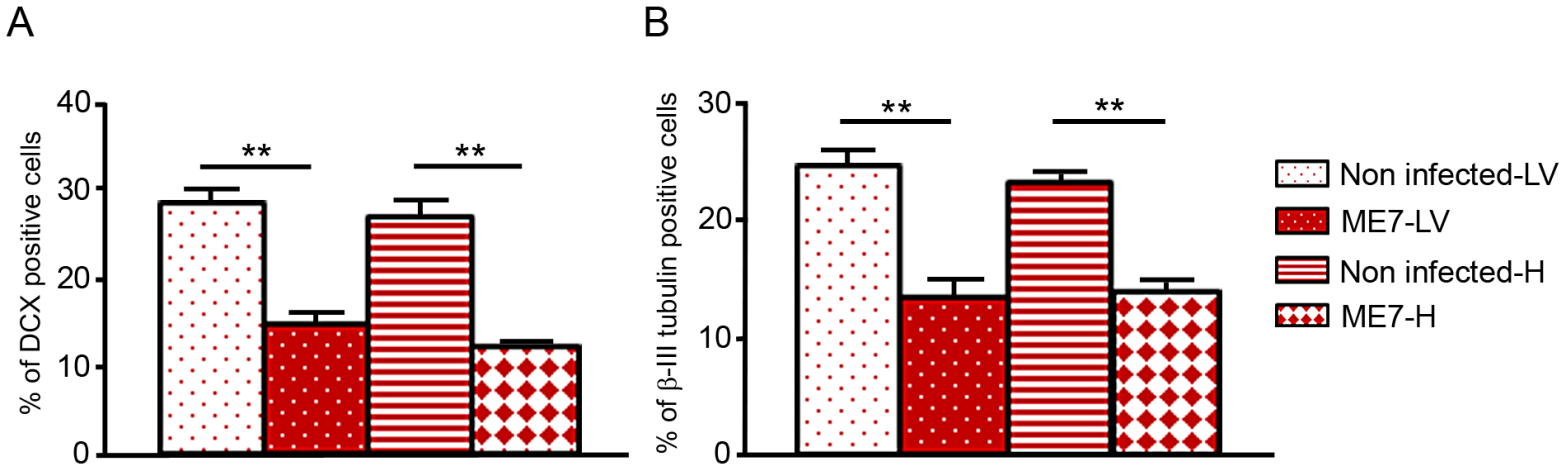
Prion infection and replication during differentiation impairs neural differentiation. Monolayer NSC derived from hippocampi and lateral ventricles were infected at the time of differentiation induction (bFGF and EGF removal) with ME7 or non infected brain homogenates as control. They were stained with anti-nestin and anti-DCX antibody 10 days after differentiation. They were also stained with anti-β-tubulin class III and anti-GFAP antibody after 10 days of differentiation. The percentage of βIII-tubulin positive cells as well as DCX positive cells were calculated with the ImageJ software. The bar graphics show higher percentages of DCX positive cells (A) as well as βIII-tubulin positive cells (B) in non-infected cells which are statistically different from percentages obtained with ME7 infected cells (p<0.0001Mann-Whitney Test). Data represent means +/− SEM from one experiment performed in triplicate. Similar results were obtained in the three independent experiments.

In order to assess whether this PrP^Sc^ accumulation impaired the neuronal differentiation process itself, NSC cells were infected just when they began their differentiation [17]. Both non infected hippocampus and lateral ventricle derived NSC were plated on poly-L-ornithine/laminin coated dishes in minimal neural N2 medium [17]. They were exposed to ME7 prion homogenate at 0.05% (p/v) at the beginning of the differentiation process (simultaneously with the EGF/bFGF withdrawal from the medium). N2 medium was completely changed after 24 hours and cells were harvested at different time points to analyse PrP^Sc^ accumulation (Figure 5). PrP^Sc^ was able to replicate in both cellular types since PrP^Sc^ was detected at 6 dpi for cells derived from the hippocampus (Figure 5A) and 8 dpi for NSC derived from the lateral ventricles (Figure 5B). Infected KOPrP cells were used as negative control of the experiment in order to detect the remaining inocula (Figure 5C). These results demonstrated that adult NSC were also capable of replicating ME7 strain during differentiation. Since there was no detectable PrP^Sc^ at day 5 of differentiation we analysed the number of neuroblasts (DCX+ cells, Figure 6A) and young neurons (bIII-tubulin+ cells Figure 6B) after 10 days of differentiation. As observed for the cells that were isolated from prion infected brain, we obtained less DCX+ cells and less βIII-tubulin cells in the infected cells than in the non-infected cells. The difference between non infected and infected cells was also statistically significant for both DCX and βIII-tubulin marker (P value<0.01, Mann-Whitney Test).

## Discussion

The presence of PrP^Sc^ among lateral ventricle NSC and neuroblasts incited us to isolate NSC cells from the brain of prion infected mice. We then checked whether these cells were infected by prion or not and assess the impact this could have on their neuronal differentiation. Our results show for the first time that NSC present in prion infected mouse brain accumulate PrP^Sc^, which leads to an alteration of their neuronal fate. Indeed, we isolated adult neural stem cells from neurogenic adult brain area (the dentate gyrus and the lateral wall of the lateral ventricles) of both prion infected and non infected mice. Importantly, we were able to keep these cells in culture for several subpassages while maintaining their prion replication. The neuronal differentiation of the infected cells was shown to be compromised since less neuroblasts and less newborn neurons were obtained when the cells were placed in a neuronal differentiation medium. This was also accompanied by an increase in the amount of astrocytes.

As neuronal differentiation impairment was also observed when the infection occurred at the beginning of the differentiation, it may suggest that some differentiation pathways are impaired when prion replicate in the cells. This is however not exclusive, with a possible additional impact of PrP^Sc^ on cell survival or on cell proliferation, but this remains to be checked in a further study. Indeed, the cellular prion protein has been shown to positively regulate neural precursor proliferation during developmental and adult mammalian neurogenesis [19], to enhance neural stem cell proliferation [20] and protect cells against oxidative stress [21], [22], [23]. A loss/alteration of PrP function could have modified the proliferative potential of the cells resulting in an inappropriate differentiation issue or apoptosis activation as it has been shown with Aβ peptides [15]. Moreover, it has been recently reported [24] that prion neurotoxicity dramatically depends on PrP^C^ expression on established neurons that also appear more susceptible to various subtoxic stimuli such as reactive oxygen species and glutamate. This study suggests also that active prion replication in neurons sensitizes them to environmental stress regulated by neighbouring cells, including astrocytes [24]. Cross-talks between astrocytes and neurons derived from the NSC from infected brains therefore represent an additional mechanism we need to investigate in further studies.

In any case, during the development of the brain pathogenesis, the compromised neurogenesis observed may probably have taken place earlier than the onset of hallmark lesions or neuronal loss. We believe that this important alteration of neurogenesis, also suggested to a lesser extent in other proteinopathies [25], represents an essential mechanism that underlies the progression and the issue of prion diseases.

Though adult neurogenesis may be beneficial for regeneration of the nervous system, our results also suggest that this system is defective during the course of the disease. While it has already been shown that neural precursor proliferation was enhanced in prion infected mice [16] the authors did not check the accumulation of PrP^Sc^ in the cells of interest. Our study is in fact the first demonstration, in a relevant prion model, of the involvement of neural stem cell in the progression of the disease. Although it also remains to be assessed, adult endogenous neural progenitors could also constitute a “reservoir” of PrP^Sc^ amplification since they can proliferate while replicating PrP^Sc^. Moreover, the fact that endogenous adult neurogenesis could be modified by the accumulation of the disease associated misfolded prion protein represents another great challenge. Inhibiting the misfolding of those pathogenic proteins would thus allow the endogenous neurogenesis to compensate injured neuronal system. Our observation regarding the status of neural stem cells during prion infection is also very important since neural stem cells graft approaches are thought to be future therapeutic strategies. This is illustrated in the case of prion diseases by one of our recent publications [26]. In this study the cell therapy approach we developed had a significant effect marked by an increase in both incubation (20.1%) and survival times (13.6%) in mice grafted before the appearance of the clinical signs. Indeed, the present results suggest that the issue of our preclinical trials would have been more successful if we had proposed a stem cell graft strategy combined with an anti-prion strategy preserving the grafted cells from prion infection and/or targeting the endogenous neural stem cells niches. This is therefore a critical issue in the search for disease-modifying therapies not only for prion disorders but also for other neurodegenerative diseases like Alzheimer or Parkinson diseases.

## Materials and Methods

Five-week-old C57Bl/6J female mice were anesthetized via intraperitoneal route with 100 µg/g of ketamine (Imalgène, Merial, Lyon, France) and 5 µg/g of xylazine (Rompun, Bayer, Leverkusen, Germany). They were then intracranially inoculated with the ME7 prion strain (1%, 20 µl) and with 1% homogenate of healthy brain as control (mock). Mice were housed in an A3 facility. Transgenic mice expressing EGFP under beta-actin promoter kindly provided by Dr. M. Okabe were also used. KO-PrP mice were kindly provided by Dr C. Weissmann. All animal work has been conducted according to relevant national guidelines of the French Ethical Committee (decree 87–848) and European Community Directive 86/609/EEC regarding mice. Experiments were performed with the approval of the Regional Languedoc Roussillon Ethical Committee for Animal Experiments under the registration number CEEA-LR-1006. They were performed in the Biohazard prevention area (A3) (Biorad/Université MontpellierII).

### Isolation of adult NSC

We have used the NeuroCult Enzymatic Dissociation Kit for Adult Stem Cell (StemCell Technologies, Grenoble, France) to isolate adult NSC from ME7 infected and non infected mice. We first dissected the lateral ventricles and hippocampus from adult mouse brains 130 days after their inoculation. They were transferred into a 100 mm dish containing NeuroCult Tissue Collection Solution. The tissue was then chopped with a scalpel for 1 minute and suspended in NeuroCult Dissociation Solution for 7 minutes at 37°C. NeuroCult Inhibition Solution was added at a 1∶1 ratio v/v and the suspension was centrifuged at 700 rpm (100× g) for 7 minutes. Pellet was then resuspended in NeuroCult Resuspension Solution. The digested tissue was mechanically dissociated by pipetting up and down 10 times, and centrifuged at 700 rpm (100× g) for 7 minutes. This step was repeated two more times with a P200 micropipettor. The final pellet was resuspended in 1 mL of Complete NeuroCult NSC Proliferation Medium (Mouse) supplemented with 20 ng/mL of recombinant human Epidermal Growth Factor and 10 ng/mL recombinant human basic Fibroblast Growth Factor (PHG0311 and PHG0021, Gibco, LifeTechnologies, Saint-Aubin, France). The cell suspension was then filtered with a 40 µm cell strainer (StemCell Technologies, Grenoble, France) and counted using Trypan Blue. Adult cells were seeded at 3.5×10^3^ cells/cm2 in 6-well tissue culture dishes (Nunc, VWR, Fontenay-sous-Bois, France).

### Differentiation assay

Under proliferation conditions, adult NSC were cultivated in T-25 cm2 tissue culture flasks (Nunc, VWR, Fontenay-sous-Bois, France). Before differentiation induction, they were first mechanically dissociated and transferred into a Poly-L-Ornithine/laminin coated dish. At 80% of confluence, cells were seeded in Poly-L-Ornithine/laminin wells of 6-well culture dishes with a cell density of 2.5×10^5^ cells/cm^2^. The day after, the NeuroCult NSC Proliferation Medium was replaced by the NeuroCult NSC Differentiation Medium (Stem Cell Technologies, Grenoble, France). This medium was replaced every 2 days and after 5 days of culture, cells were fixed with 4% paraformaldehyde for 15 minutes at room temperature.

### Infection of NSC during differentiation

Neurospheres were transferred onto Poly-L-Ornithine/laminin coated 6-well plates (Nunc) in N2 medium with a cell density of 2.5×10^5^ cells/cm^2^. They were maintained on monolayer for several subpassages to adapt the cells to the new conditions. When the culture reached the 80–90% of density, 0.05% (p/v) of ME7 or healthy brain homogenate were added into the N2 medium. Cells were incubated in the presence of the inocula for 24 h. The culture was rinsed twice and fresh N2 medium was added. Media were replaced every 2 days. To monitor any remaining inocula, KOPrP NSC cells were used as control.

### Immunofluorescence assay for NSC markers

Cells were permeabilized with 0.1% triton X-100 in PBS during 3 minutes, washed with PBS-BSA 0.2% three times. Saturation was performed with PBS-0.2%BSA for 1 hour at room temperature. Cells were then incubated with the primary antibodies (Nestin (Chemicon, MerckMillipore, Billereca, USA), DCX (Abcam, Paris, France), GFAP (Dako, Trappes, France), and beta-III tubulin (TujI clone, Covance, Rueil Malmaison, France), 1∶500 in PBS-0.2%BSA) for 1 hour at 37°C. Cells were then washed with PBS-0.2%BSA and were incubated with the appropriate secondary antibodies (goat anti-rabbit-Alexa fluor 488 and goat anti-mouse-Alexa fluor 555 (Invitrogen LifeTechnologies, Saint-Aubin, France), 1∶7000) for 1 hour at room temperature. After sequential washes with PBS-0.2%BSA, nuclei were stained with Hoechst 33258 (Calbiochem, VWR, Fontenay-sous-Bois, France) for 5 minutes under agitation at room temperature and then rinsed with PBS and H2O. The slides were mounted using the FluorSave Reagent mounting medium (Calbiochem, VWR, Fontenay-sous-Bois, France). Photos were taken with a Leica DMRA2 microscope and ImageJ software was used to count the cells. For the statistical analysis we used the Mann-Whitney test. For each condition, images were acquired from 5 to 8 fields and the experiment was repeated three times. An average of 80 cells was counted in each field.

### Immunofluorescence assay for PrP^Sc^ analysis

Cells were permeabilized with 0.5% triton X-100 in PBS during 5 minutes and washed with PBS-BSA 0.2% three times. PrP^Sc^ epitope retrieval was obtained using 3M guanidium thiocyanate/PBS during 5 minutes and washed with PBS-BSA 0.2% and PBS. The saturation was then obtained with PBS-0.2%BSA for 1 hour at room temperature. The cells were incubated with the primary antibody SAF61 (1∶300) in PBS-5% Milk over night at 4°C. The remaining steps were the same as described before [27].

### PrP^Sc^ Western blot analysis

PrP^Sc^ presence was checked by western blot analysis using the saf Mix anti-PrP cocktail (saf 60, saf 69 and saf 70 antibodies) as described elsewhere [27].

### Preparation of brains for histological analysis

Mice were anesthetized as described above and then perfused with paraformaldehyde 4%. The brains of the mice were collected and placed in paraformaldehyde 4% for 24 hours at 4°C. They were then manually embedded in paraffin (Paraplast, Microm, Villefranche sur Saone, France) and cut in sections of 5 µm thick using a Leica microtome. The sections were collected on microscope slides without treatment (Starfrost, Microm, Villefranche sur Saone, France).

### Immunohistochemistry

Tissues were dewaxed using Clearify solution (Microm, Villefranche sur Saone, France) and then rehydrated with decreasing degrees of ethanol washes.

Nestin and DCX immunohistochemistry: Sections were incubated in H2O2 0.5% for 20 minutes at room temperature and washed with H2O and PBS Epitope retrieval was performed by heathing the sections in 0.1 M EDTA. Sections were then saturated with PBS-0.1%BSA-0.1%Triton X-100 for 1 hour and then incubated overnight at 4°C with the pre-diluted anti-Nestin (Chemicon, MerckMillipore, Billereca, USA) primary antibody or anti-DCX primary antibody (1/300, Abcam, Paris, France). The secondary antibody used was a biotinylated goat anti-mouse or anti-rabbit antibody (Amersham, Velizy-Villacoublay, France) (1∶1000 in PBS-0.1%triton X-100). The avidin-peroxidase complex (Vectastain Elite kit, Vector laboratories, Clinisciences, Nanterre, France) was then added and then revealed with 3,3′-diaminobenzidine (DAB) (Vector laboratoriess, Clinisciences, Nanterre, France), according to the manufacturers' instructions.

PrP^Sc^ immunohistochemistry: PrP^Sc^ was analysed by immunohistochemistry using the Saf84 (0.5 µg/ml) anti-PrP antibody [28], [29]. SAF84 monoclonal antibody recognising the human 161–170 PrP sequence was kindly provided by Dr J. Grassi (CEA/SPI, Saclay, France). For PrP^Sc^ immunostaining, epitope retrieval consists in a treatment with formic acid (10 minutes) followed by an autoclaving treatments (121°C, 10 minutes). The secondary antibody used was a biotinylated goat anti-mouse antibody (Amersham, Velizy-Villacoublay, France) (1∶1000 in PBS-0.1%triton X-100). The avidin-peroxidase complex (Vectastain Elite kit, Vector laboratories, Clinisciences, Nanterre, France) was then added and then revealed with 3,3′-diaminobenzidine (DAB).

For the double immunostaining procedure, we proceeded as described in [30]. Briefly, we first performed the protocol described above for nestin or DCX immunostaining using EDTA epitope retrieval pretreatments. The slides were then revealed using DAB. The brown precipitate given by DAB resists to PrP^Sc^ specific pretreatments (formic acid and autoclave). Then the slides were treated according to the procedure described above for PrP^Sc^ immunostaining (formic acid and autoclave). PrP^Sc^ revelation was performed using histogreen kits giving a blue green coloration.

### Accession numbers/ID numbers

List of the accession numbers for genes and proteins mentioned in the text (UniProt):

PrP: P04925

Nestin: Q6P5H2

Doublecortin: O88809

Beta-III-tubulin: Q9ERD7

Glial fibrillary acidic protein: P03995
